# Supplementary figures and images for: Cross-Species Transmission and Differential Fate of an Endogenous Retrovirus in Three Mammal Lineages
Source: PLoS Pathog. 2015 Nov 12;11(11):e1005279. doi: 10.1371/journal.ppat.1005279 (PMC4643047; doi:10.1371/journal.ppat.1005279)

1000.0

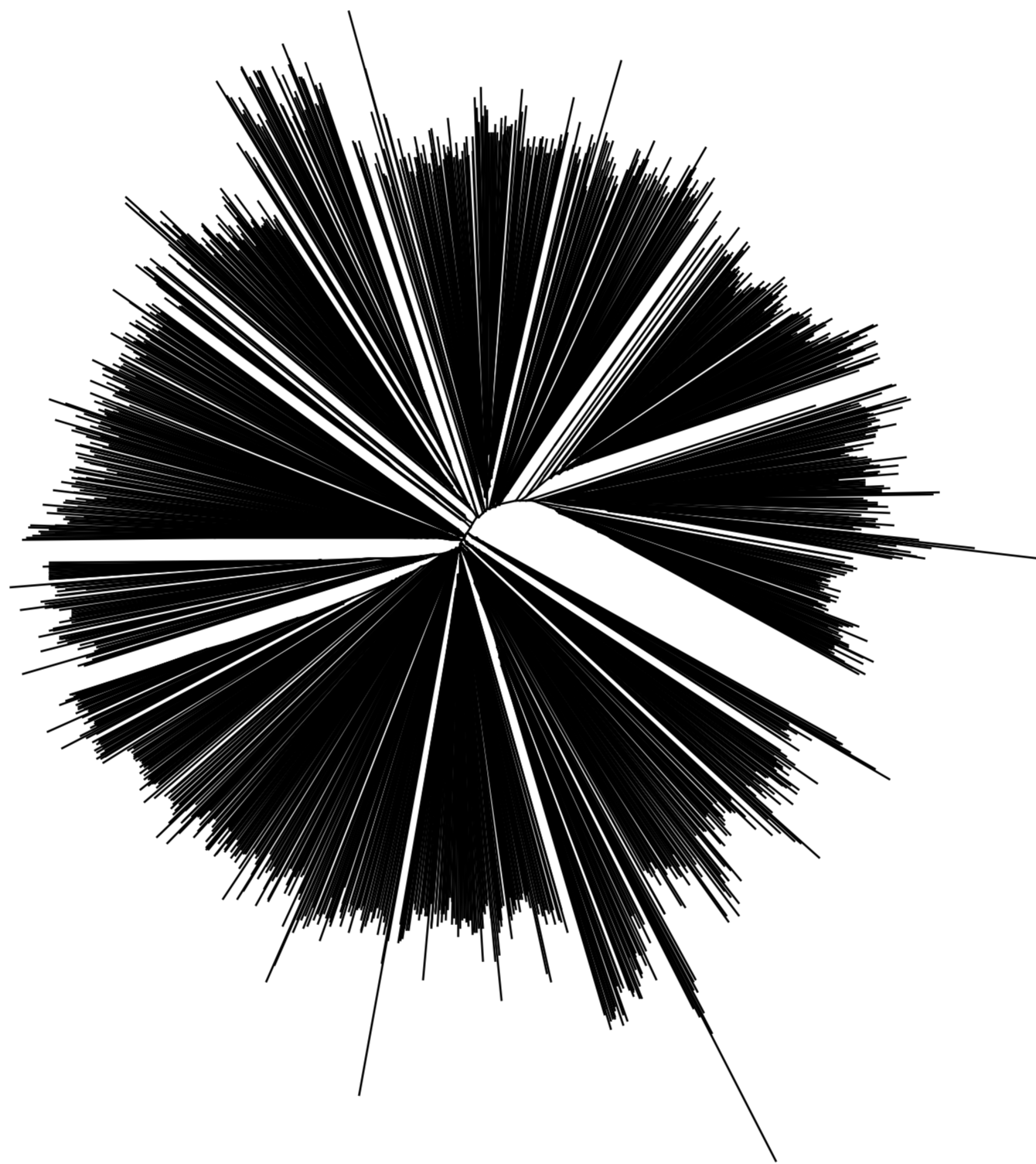

Supplement: S1 Fig — (PDF) [file ppat.1005279.s002.pdf]

0.1

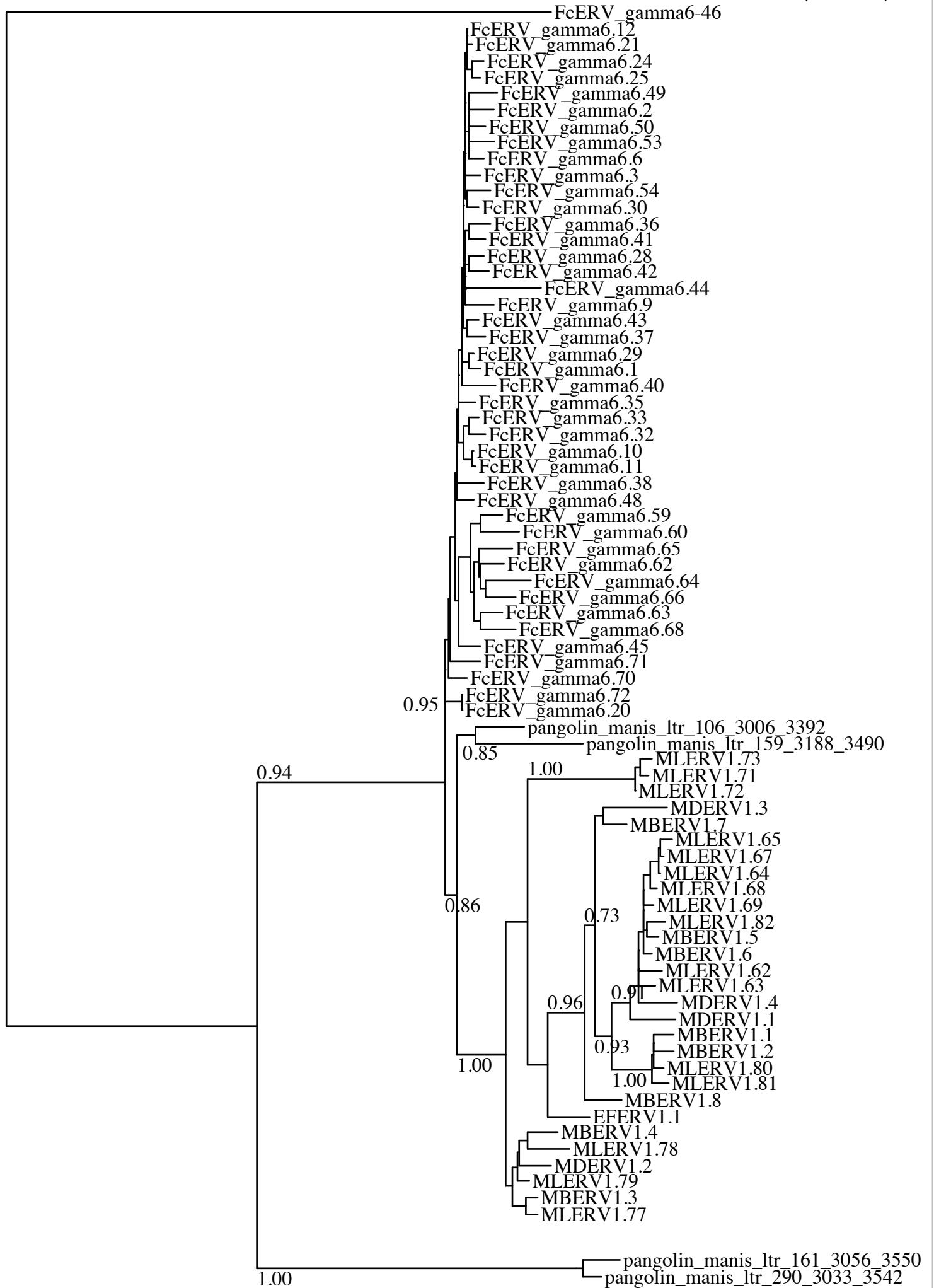

Supplement: S3 Fig — (PDF) [file ppat.1005279.s004.pdf]

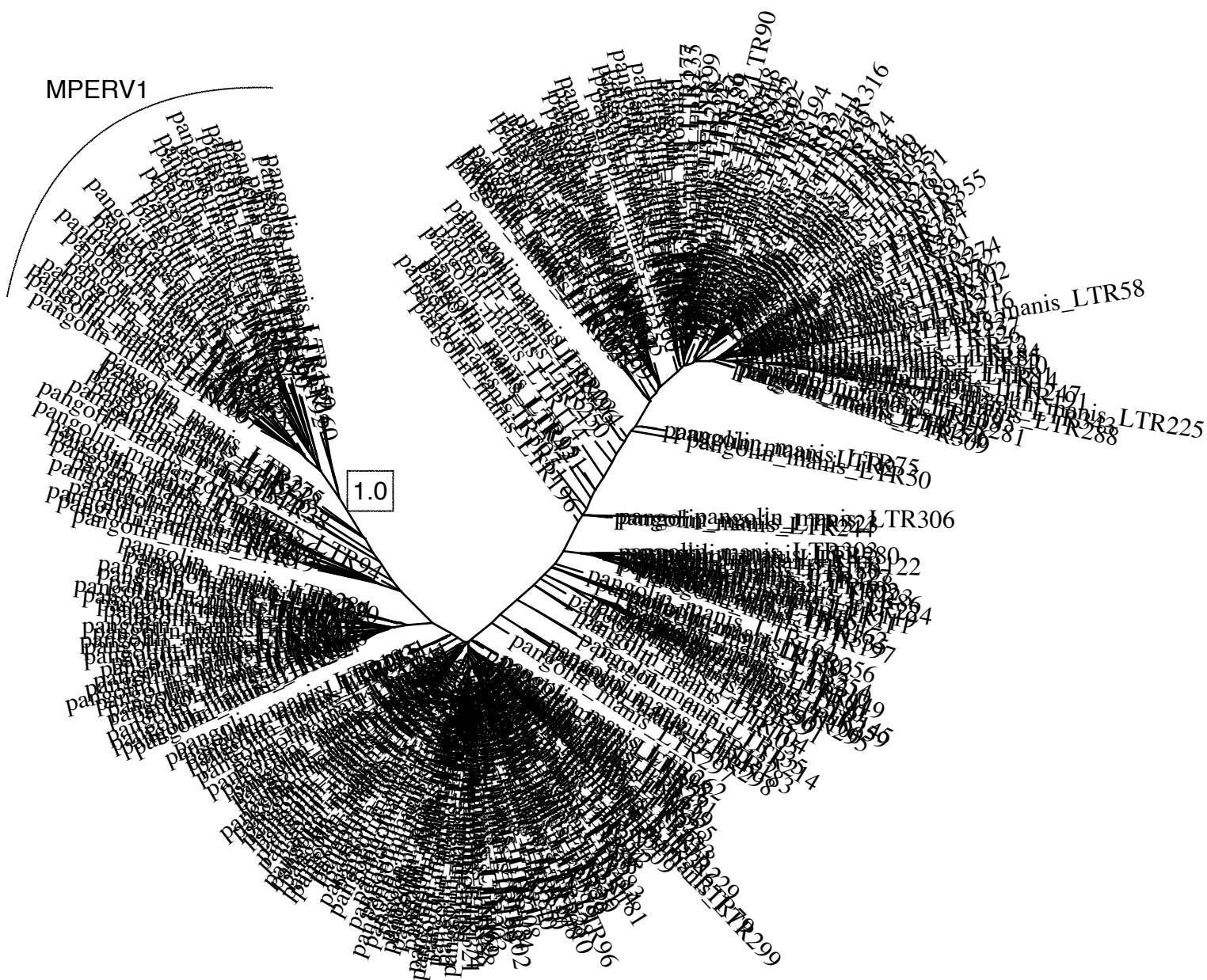

Supplement: S5 Fig — All members of MPERV1 are enclosed within an arch, and the aLRT support of MPERV1 is labeled at the branch node. (PDF) [file ppat.1005279.s006.pdf]

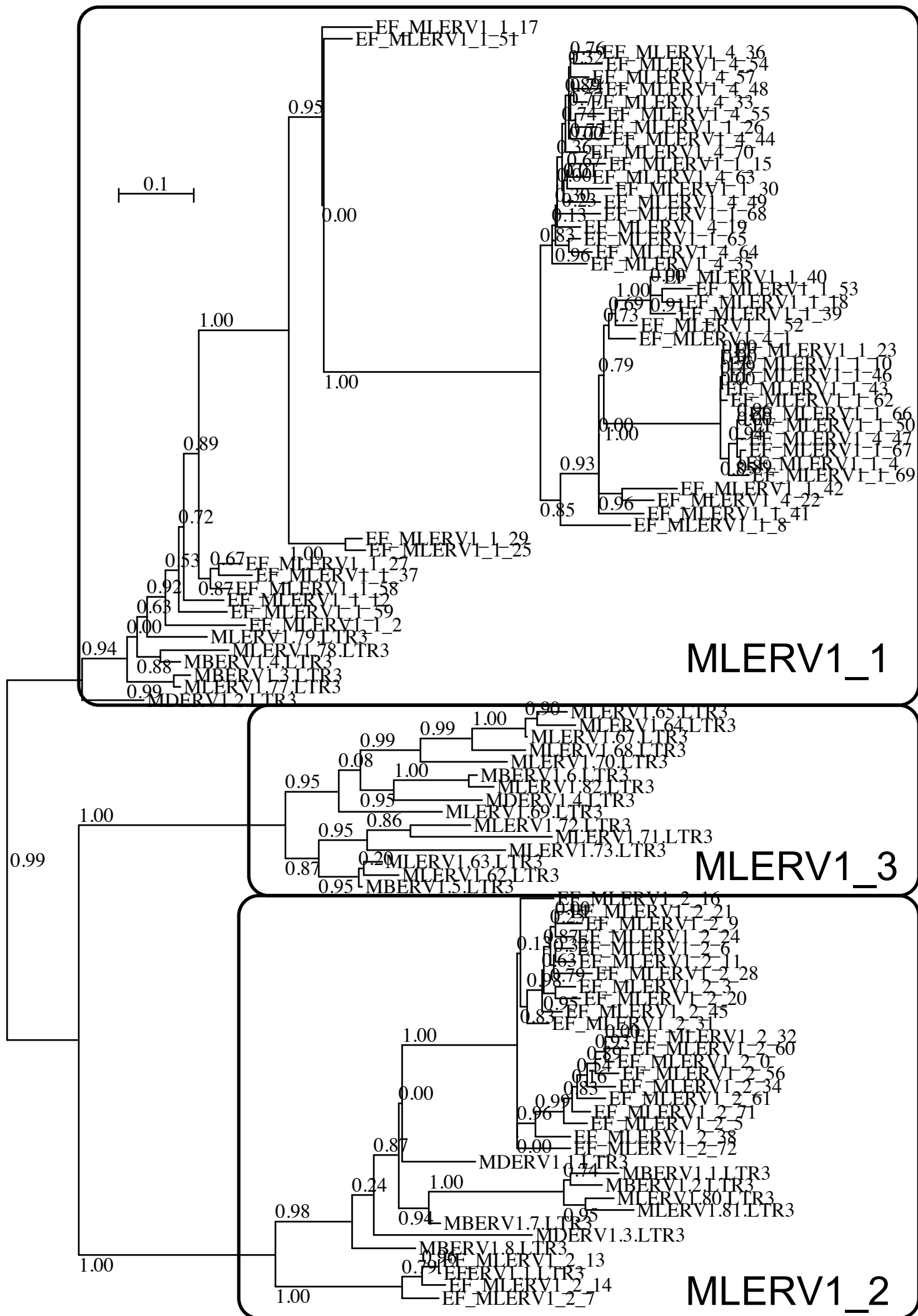

Supplement: S6 Fig — Three subfamilies of MLERV1 are illustrated. SoloLTRs in E. fuscus cluster with either MLERV1_1 or MLERV1_2. (PDF) [file ppat.1005279.s007.pdf]
