# Supplementary material for: Cross-Species Transmission and Differential Fate of an Endogenous Retrovirus in Three Mammal Lineages
Source: PLoS Pathog. 2015 Nov 12;11(11):e1005279. doi: 10.1371/journal.ppat.1005279 (PMC4643047; doi:10.1371/journal.ppat.1005279)
Supplement: S4 Fig — Y axis represents frequency of stop codon or frameshift indel per codon in each domain, four different subfamilies are illustrated with different colors. Error bars represent 95% confidence interval. (PDF) [file ppat.1005279.s005.pdf]

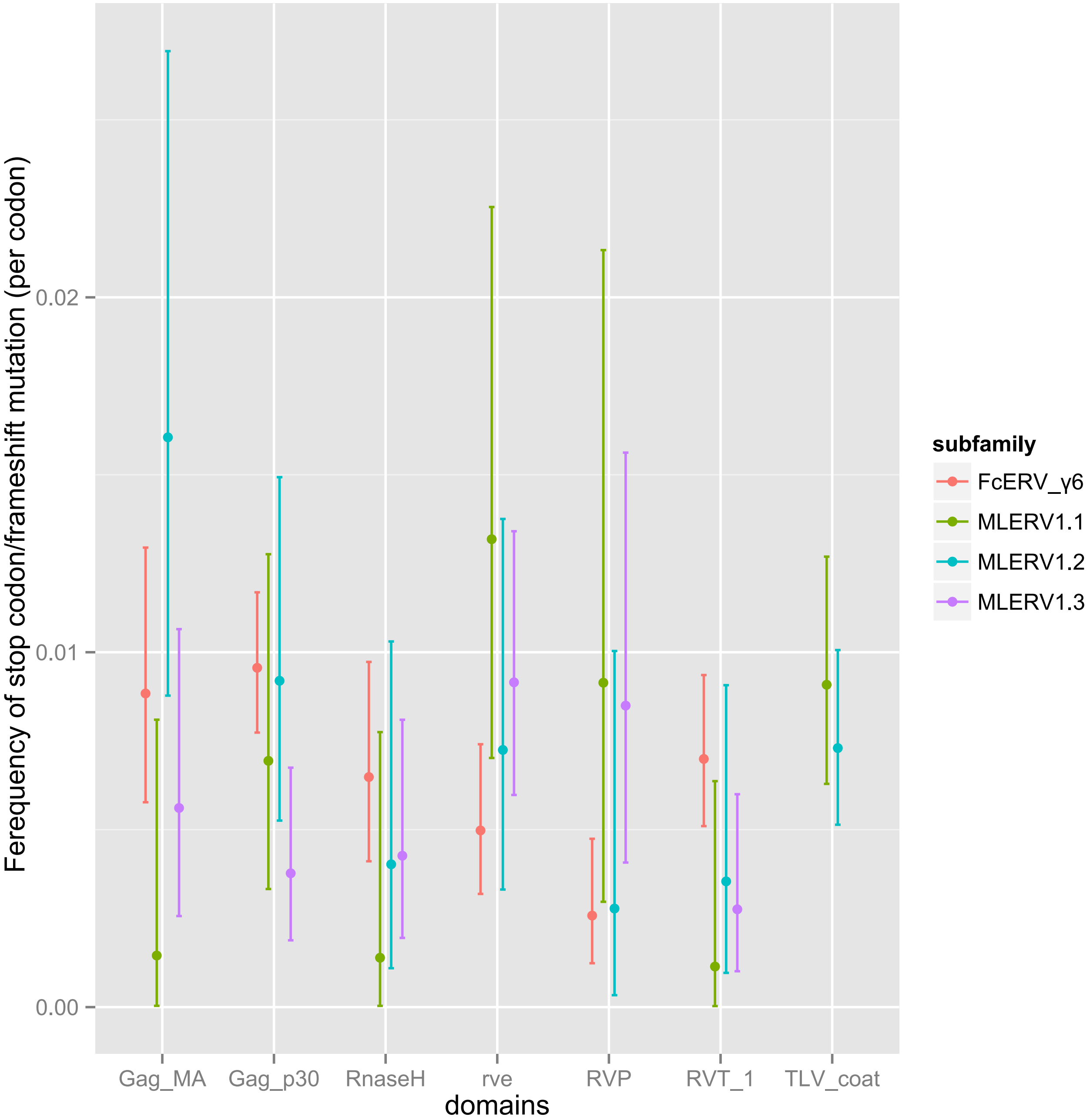

Ferequency of stop codon/frameshift mutation (per codon)
0.02
0.01
0.00
Gag_MA
Gag_p30
RnaseH
rve
RVP
RVT_1
TLV_coat
domains
subfamily
FcERV_γ6
MLERV1.1
MLERV1.2
MLERV1.3
