# Supplementary material for: Cross-Species Transmission and Differential Fate of an Endogenous Retrovirus in Three Mammal Lineages
Source: PLoS Pathog. 2015 Nov 12;11(11):e1005279. doi: 10.1371/journal.ppat.1005279 (PMC4643047; doi:10.1371/journal.ppat.1005279)

LTR_align_webPRANK.CpG.new Tue May 19 16:06:04 2015

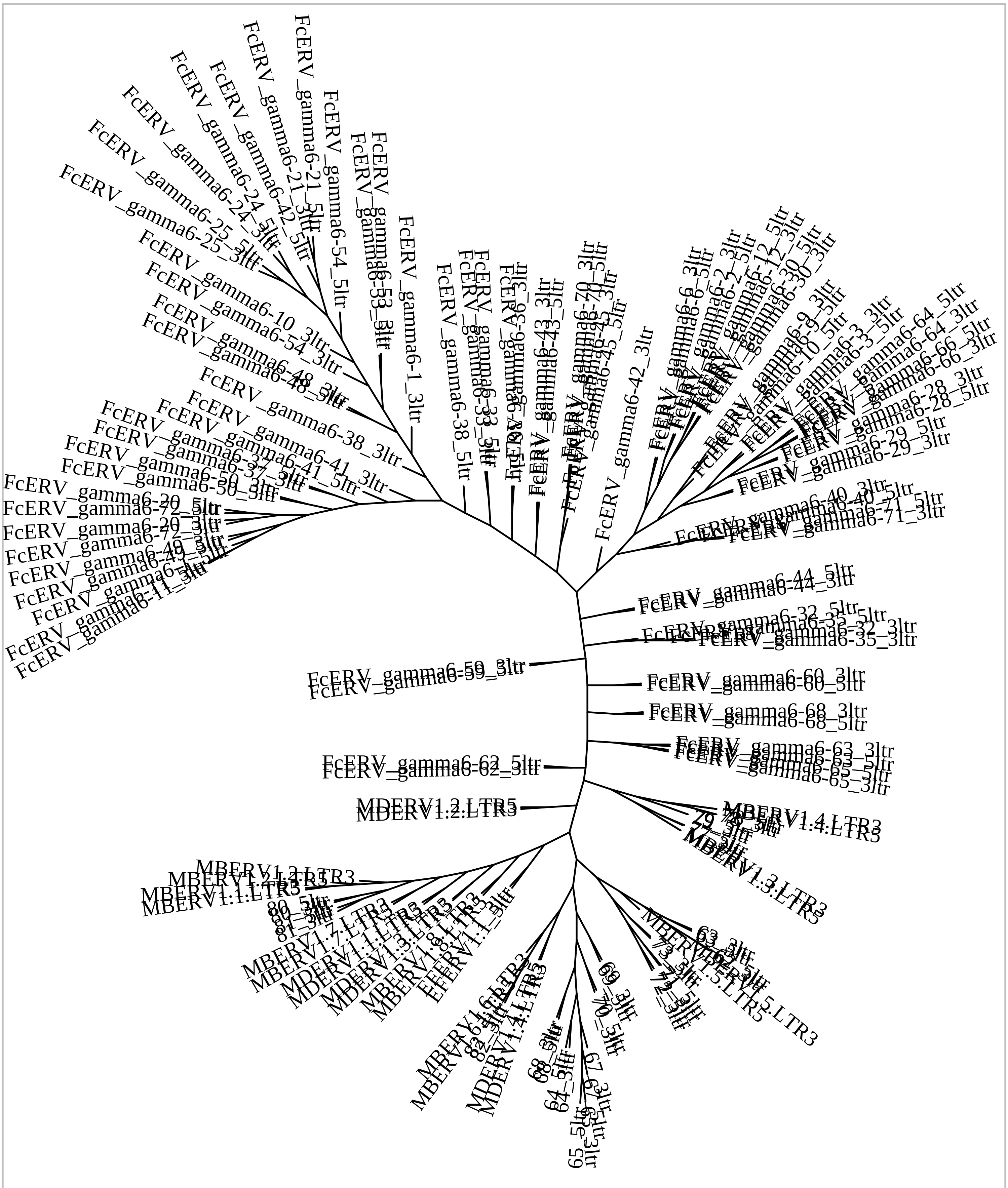

Supplement: S2 Fig — (PDF) [file ppat.1005279.s003.pdf]
